# Potential diagnostic biomarkers for chronic kidney disease of unknown etiology (CKDu) in Sri Lanka: a pilot study

**DOI:** 10.1186/s12882-017-0440-x

**Published:** 2017-01-19

**Authors:** Saravanabavan Sayanthooran, Dhammika N. Magana-Arachchi, Lishanthe Gunerathne, Tilak Abeysekera

**Affiliations:** 1 Cell Biology Group, National Institute of Fundamental Studies, Kandy, Sri Lanka; 2 Renal Care & Research Centre, District Hospital, Girandurukotte, Sri Lanka; 3 Department of Pharmacology, Faculty of Medicine, University of Peradeniya, Kandy, Sri Lanka

**Keywords:** Gene expression analysis, Kidney injury, Oxidative stress, RT-qPCR

## Abstract

**Background:**

In Sri Lanka, there exists chronic kidney disease of both known (CKD) and unknown etiologies (CKDu). Identification of novel biomarkers that are customized to the specific causative factors would lead to early diagnosis and clearer prognosis of the diseases. This study aimed to find genetic biomarkers in blood to distinguish and identify CKDu from CKD as well as healthy populations from CKDu endemic and non-endemic areas of Sri Lanka.

**Methods:**

The expression patterns of a selected panel of 12 potential genetic biomarkers were analyzed in blood using RT-qPCR. Fold changes of gene expressions in early and late stages of CKD and CKDu patients, and an apparently healthy population of a CKDu endemic area, Girandurukotte (GH) were calculated relative to apparently healthy volunteers from a CKDu non-endemic area, Kandy (KH) of Sri Lanka, using the comparative CT method.

**Results:**

Significant differences were observed between KH and early stage CKDu for both the insulin-like growth factor binding protein 1 (IGFBP1; *p* = 0.012) and kidney injury molecule-1 (KIM1; *p* = 0.003) genes, and KH and late stage CKD and CKDu for the glutathione-S-transferase mu 1 (GSTM1; *p* < 0.05) gene. IGFBP1 and KIM1 genes showed significant difference between the early and late stage CKDu (*p* < 0.01). The glutamate cysteine ligase catalytic subunit (GCLC) gene had significantly different expression between KH and all the other study groups (*p* < 0.01). The GH group was significantly different from the KH group for the oxidative stress related genes, G6PD, GCLC and GSTM1 (*p* < 0.01), and also the KIM1 gene (*p* = 0.003). IGFBP1, insulin-like growth factor binding protein 3 (IGFBP3), fibronectin 1 (FN1) and KIM1 showed significant correlations with serum creatinine, and IGFBP1, KIM1 and kallikrein 1 (KLK1) with eGFR (*p* < 0.05).

**Conclusion:**

A panel consisting of IGFBP1, KIM1, GCLC and GSTM1 genes could be used in combination for early screening of CKDu, whereas these genes in addition with FN1, IGFBP3 and KLK1 could be used to monitor progression of CKDu. The regulation of these genes has to be studied on larger populations to validate their efficiency for further clinical use.

## Background

Chronic kidney disease (CKD) is increasing rapidly worldwide and is gaining much attention in both the developed as well as developing countries. CKD is characterized by a reduced glomerular filtration rate (GFR) that is accompanied with structural or functional abnormalities of the kidneys on urinalysis, biopsy and imaging [1].

The concept of biomarker discovery in medicine is becoming increasingly important due to its potential for early screening, more effective treatment and a more personalized approach to medical care [2].“A biological marker (biomarker) is a characteristic that is objectively measured and evaluated as an indicator of normal biological processes, pathogenic processes, or pharmacologic responses to a therapeutic intervention.”[3] The biomarkers could be identified at any level along the genome – phenome continuum, and could be genomic biomarkers (DNA or RNA), proteomic biomarkers (proteins) or metabolic biomarker (metabolites) [3].

Biomarkers in both acute kidney injury (AKI) and CKD look for similar effects of the diseases; decrease in nephron number, vascular insufficiency, and cell cycle disruption [1]. The biomarkers of kidney disease should however be complemented with clinical assessments of patients with CKD and AKI and not be used in isolation [1]. The intended biomarkers should provide rapid, non-invasive and specific measurements that correlate well with kidney tissue pathology [1].

The most sensitive marker of CKD progression in clinical practice is proteinuria, especially when combined with the estimated glomerular filtration rate (eGFR) [2]; however, the process of kidney injury starts with the induction of molecular level changes, which therefore gives promise for identification of molecular markers for early diagnosis of disease process. Biomarkers are needed in CKD to help estimate GFR, assess cardiovascular disease, determine metabolic abnormalities associated with CKD and differentiate inflammatory and fibrotic conditions of the kidney [1]. The etiologies for CKD differ and therefore comorbidities exist in most CKD patients, hence a single biomarker would be incapable of satisfying all these needs [1, 2].

In Sri Lanka, there exists CKD of known etiologies (with the majority being diabetes and cardiovascular diseases) and unknown etiology (CKDu). This CKDu is confined to certain geographical locations of the country, notably the dry zones, in the North Central Province and Uva Province where the majority of the population are involved in farming as the main occupation. The majority of the hypotheses for the cause of the disease revolve around environmental stimuli based on the environmental location and occupation of the affected individuals [4–9]. The main biomarkers for screening and identification of CKDu include dipstick proteinuria, serum creatinine and eGFR measurements, while excluding known etiologies of CKD such as diabetes, hypertension, and systemic lupus erythematosus [10]. The presence of small, echogenic kidneys, and tubulointerstitial damage on renal biopsy further confirm diagnosis [11]. As with CKD, the identification of diseased individuals takes place only in the later stages of the disease where the symptoms become apparent.

Although analyzing renal tissue samples would be most ideal as it is the affected tissue, the obtaining of biopsies is invasive, more expensive and not suitable for initial screening purposes [12]. This brings about a need for other suitable tissues for the safe, reliable and inexpensive monitoring of biomarkers. Body fluids like serum and urine are mainly targeted for the identification of biomarkers as they are the least invasive methods and replace the usage of tissue biopsies [13].

In this pilot study, genes that have potential as screening and prognostic biomarkers of CKD, both known and unknown etiology, were selected from literature to cover more than one particular characteristic/function related to the disease. The expression pattern of selected genes, namely kidney injury molecule-1 (KIM1), fibronectin 1 (FN1), insulin like growth factor binding protein 1 (IGFBP1), insulin like growth factor binding protein 3 (IGFBP3), kallikrein 1 (KLK1), glutathione S transferase mu 1 (GSTM1), glutamate cysteine ligase catalytic subunit (GCLC), glucose-6-phosphate dehydrogenase (G6PD), cytochrome P450 enzyme 2D6 (CYP2D6) and 2C19 (CYP2C19) were analyzed in both chronic kidney disease patients of known and unknown etiology residing in Girandurukotte, a CKDu endemic area belonging to the dry zone of Sri Lanka. The majority of cases in this region belong to the CKDu category. Healthy individuals from the same area who are considered at risk and have been previously screened for CKDu and found negative, and healthy individuals from an area not endemic for CKDu, Kandy, located in the Central Province, wet zone of the country were selected as controls.

Among the genes selected, two were related to kidney injury and repair, KIM1 and FN1. KIM1 is a novel biomarker of kidney injury that is upregulated in dedifferentiated proximal tubule epithelial cells in kidney after ischemic or toxic injury [14]. FN1 is a glycoprotein involved in cell adhesion and migration process including wound healing, blood coagulation and host defense. It has also been linked to tissue scarring and fibrosis [15].

Metabolic disorders such as diabetes and cardiovascular diseases are either causative factors or complications of chronic kidney diseases. Therefore three genes related to cardiovascular complications and diabetes were selected to observe their use as possible prognostic biomarkers. IGFBP1and IGFBP3 belong to a family of IGFBPs which has six proteins that specifically bind insulin like growth factors I (IGF-I) and II (IGF-II). The IGF system has been increasingly implicated in the development of cardiovascular diseases [16]. They are multifunctional as they not only passively circulate transporters, but also play a variety of roles in the circulation, extracellular environment, and inside the cell [17]. IGFBP3 has the major IGF transport function and is the most abundant circulating IGFBP. It has been suggested to have a role of wound healing by binding to the IGF-1 and releasing them at the wound sites [17].

KLK1 belongs to the kallikreins, a different group of proteins, serine proteases that have been related to human essential hypertension and associated complications. Low levels of urinary kallikreins have been associated with hypertension and renal disease [18].

There have been hypotheses suggesting the possible role of metal toxicity and environmental toxins as etiological factors of CKDu in Sri Lanka [19]. We therefore selected genes related to metal toxicity and oxidative stress (GSTM1, GCLC and G6PD), and xenobiotic metabolism (CYP2D6 and CYP2C19) which could be possibly influenced by the environmental toxins, in turn acting as biomarkers of toxicity.

Glutathione (GSH) plays a crucial role in the antioxidant defense system and has a predominant role in the regulation of the intracellular redox state and protects cells from oxidative injury [20]. Two genes related to GSH were selected to be studied; GCLC, the rate limiting enzyme in the production of GSH, and GSTM1, the enzyme that facilitates the detoxification action of GSH by providing a hydrophilic binding site for glutathione and a hydrophobic binding site for electrophilic substrates [21]. The G6PD enzyme is another protein that enables cells to counterbalance the oxidative stress via the activation of the glutathione system by the production of nicotinamide adenine dinucleotide phosphate (NADPH) [22].

Two genes belonging to the cytochrome P450 monoxygenase enzyme family, CYP2D6 and CYP2C19 were selected to study any differential expression influenced by drugs or xenobiotics. CYP2D6 metabolizes 20–25% of the clinically used drugs [23], whereas CYP2C19 metabolizes 8% of all drugs [24]. Such drug metabolizing enzymes are not only expressed in liver tissue, but also extra hepatic tissues like blood lymphocytes, kidney and intestine [25]. Studies have shown that substrate metabolism and toxicity can be influenced by the xenobiotic modulation of the CYP genes and that they could hence be sensitive markers of chemical exposure [26, 27].

The expression patterns of the genes were tested in the CKD, CKDu and apparently healthy, risk groups of the CKDu endemic area in comparison to apparently healthy individuals of a non-endemic area in order to identify possible early screening and prognostic markers.

## Methods

### Patients

The patients to be studied were recruited from June 2013 to November 2015 from the Renal Care & Research Centre, District Hospital, Girandurukotte, a region endemic for CKDu, located in the Uva Province, belonging to the dry zone of Sri Lanka. The diagnosis of patients was carried out by the nephrologist attending the Renal Clinic of the hospital.

CKDu was labeled as having unknown etiology based on criteria set by the Ministry of Health, Sri Lanka with no past history of diabetes mellitus, chronic or severe hypertension, snake bite, glomerulonephritis or urological diseases being causes for the disease. A normal HBA1C (<6.5%), blood pressure <160/100 mmHg untreated or <140/90 mmHg on up to two antihypertensive medications were additional features. The stages of CKD/CKDu are classified according to the GFR levels (Table 1) [28]. The serum creatinine is also used to estimate the GFR (eGFR) levels [29] and therefore is an important biomarker of kidney injury. The equation used was that proposed by Levey et al. [30] (Eq. 1).

**Table 1:** Different stages of chronic kidney disease classified by glomerular filtration rates

Stage of chronic kidney disease	Glomerular filtration rate (mL/min/1.73 m^3^)
Stage 1	≥90
Stage 2	60–89
Stage 3	30–59
Stage 4	15–29
Stage 5	<15 (or dialysis)

1 $$ \mathrm{eGFR} = 186 \times {\left[\mathrm{serum}\ \mathrm{creatinine}\ \left(\mathrm{mg}/\mathrm{dL}\right)\right]}^{-1.154} \times {\left(\mathrm{age}\right)}^{-0.203} \times \left(0.742\ \mathrm{if}\ \mathrm{female}\right) $$

The patient population was categorized into four categories; early stage CKDu (*n* = 11), late stage CKDu (*n* = 23), early stage CKD(*n* = 5) and late stage CKD (*n* = 9), where the early stages consisted of stage 1 to stage 3 patients and late stages consisted of stage 4 and stage 5 patients.

### Healthy volunteers

Two apparently healthy groups were selected for the study, consisting of volunteers from both the CKDu endemic region, Girandurukotte (GH; *n* = 5), located in the Uva Province, dry zone of the country, as well as a region not endemic to CKDu, Kandy (KH; *n* = 7), located in the Central Province, wet zone of the country. The residents of Girandurukotte undergo routine screening for CKD/u and the selected individuals were not identified as being diseased. However, the individuals of the Girandurukotte healthy population, being of the same environmental area, are considered to be under risk, due to causative factors of CKDu being hypothesized as environmental related and therefore their expression patterns were also analyzed with respect to the healthy group from Kandy.

### Sample collection and storage

Blood samples were collected during routine blood collection from patients. Whole blood (1 mL) was collected into 3 mL TRIzol LS reagent (Invitrogen), shaken vigorously to ensure lysis and transported in ice from the District Hospital, Girandurukotte to the National Institute of Fundamental Studies, Kandy, where it was stored under −20 °C. RNA extractions were carried out the day following arrival at the facility.

### RNA extraction and RT-qPCR

RNA was extracted according to the TRIzol LS (Invitrogen) manufacturer’s protocol followed by purification using PAXgene spin columns (PreAnalytix). All extracted RNA samples were checked for integrity by using Agarose gel electrophoresis, and looking for 28 s:18 s rRNA intensity of approximately 2:1. The absorbance values, A280/A260 and A230/A260 were measured using UV spectrophotometry (Shimadzu) to interpret protein and salt impurities and values had to be greater than 1.7 and 1.0 respectively for inclusion in study. A total of 1 μg of RNA as measured by the QuantiFluor-ST RNA system (Promega) was used in the preparation of cDNA (Quantitect Reverse Transcription Kit, Qiagen). Equal amount of cDNA (total RNA = 50 ng) was used in each PCR reaction.

PCR Mastermix was prepared using the Quantitect Probe PCR Kit (Qiagen) and reaction components included 10 μL QuantiTect Probe PCR mastermix (Qiagen), 4 μL RNase free H_2_O, 0.8 μM forward and reverse primers, 0.2 μM probe and 50 ng of cDNA template. The cycler was programmed for initial activation of HotStartTaq polymerase at 95 °C for 15 mins, and 50 cycles of denaturation at 95 °C, 30s and combined annealing and extension at 60 °C, 60s. Hydrolysis probes were used for the fluorescent detection and quantification of PCR amplification. Self designed as well as pre designed primers were used for the PCR amplification [31–35]. Details of primers and probes used have been presented in Table 2. The fluorescence for probe detection was acquired in the extension steps using the green channel of the RotorGene Q cycler (Qiagen).

**Table 2:** Details of primer and probe sequences

Name	Accession Number	Sequences 5’–3’ (F- forward, R- reverse, P- probe)
GCLC	NM_001498.3	F: ACAAGGACGTTCTCAAGTG R: AGGATGGTTTGGGTTTGTC P: CCTGTCTGGGGAGAAAGTTCTTGAAACTCT
KIM1	NM_012206.2	F: AACCAGTAGCCACTTCAC R: CTGTCACGGTGTCATTCC P: TCAGCCAGCAGAAACCCACCC
GSTM1	NM_000561.3	F: GCATGATCTGCTACAATCCA R: TTGTTTCCTGCAAACCAT P: CCTGAAAAGCTAAAGCTCTACTCAGAG
KLK1	NM_002257.3	F: GGACTACAGCCACGACCTCATGCTGC R: GTCCACACACTGGAGATCATCTGG P: TGGAGTTGCCCACCGAGGAACCCGAA
CYP2D6	NM_000106.5	F: TAGTGGTGGCTGACCTGTTCTCT R: TCGTCGATCTCCTGTTGGACA P: CTCCTGCTCATGATCCTACATCCGGA
CYP2C19	NM_000769.1	F: GAACACCAAGAATCGATGGACA R: TCAGCAGGAGAAGGAGAGCATA P: TAATCACTGCAGCTGACTTACTTGGAGCTGGG
IGFBP1	NM_000596.2	F: GGGACGCCATCAGTACC R: CCATTTTTTGATGTTGGTGAC P: ATGATGGCTCGAAGGCTCTCCA
IGFBP3	NM_000598.4	F: CAGAGCACAGATACCCAGAACTTC R: TTCTCTACGGCAGGGACCAT P: ATTCTGTCTCCCGCTTGGACTCGGA
FN1	NM_212482.1	F: GAAGAGCGAGCCCCTGAT R: GGGGTCTTTTGAACTGTGGA P: AGACGAGCTTCCCCAACTGGTAACCCCTT
G6PD	NM_000402.4	F: TGCCCCCGACCGTCTAC R: ATGCGGTTCCAGCCTATCTG P: ACTCGTGAATGTTCTTGGTGACGGCC
B2M	NM_004048.2	F: TGCCGTGTGAACCATGTGA R: CCAAATGCGGCATCTTCAA P: TGATGCTGCTTACATGTCTCGATCCCACT
GAPDH	NM_001289745.1	F: TGACCTCAACTACATGGTTTA R: GCCCCACTTGATTTTGGA P: CCATGGCACCGTCAAGGCTGA

### Quantification of fold changes

Fold changes were calculated using the comparative Ct method [36] with two reference genes, B2M and GAPDH. The KH group was used for calibration. The individual fold changes were calculated and geometric means obtained of the respective groups.

### Statistical analysis

Log 2 normalized values of the fold changes were used for further statistical analysis. One way ANOVA and post hoc Games-Howell analysis were carried out to identify significant results. Outliers, extreme of 1.5 times of inter-quartile range (IQR), calculated separately for each study group were excluded in ANOVA calculations. The log 2 normalized fold changes were also analyzed for correlation with the currently used marker of CKD, serum creatinine, using the Pearson correlation coefficient and two tailed significance values. Missing values were replaced with means for the correlation calculations. The correlations were carried out separately for the CKD and CKDu groups.

Gender-wise difference in expression of the genes was analyzed using two-tailed ttest in between the groups.

## Results

### Study population

The characteristics of the study population are summarized in Table 3. A total of 60 subjects were included in the study; 51 males and 9 females. The mean age of the study population was 51 ± 12. The serum creatinine and eGFR levels of the early stage CKDu were 1.62 ± 0.74 and 52.03 ± 22.52, and the late stage CKDu were 4.03 ± 2.52 and 21.61 ± 11.03 respectively. The serum creatinine and eGFR levels of the early stage CKD were 1.28 ± 0.45 and 64.26 ± 21.25, and the late stage CKD were 3.72 ± 1.36 and 20.16 ± 7.46 respectively. The healthy populations from both the Girandurukotte as well as Kandy areas were selected based on no current illnesses and no previous records of chronic illnesses; their serum creatinine and eGFR levels were not measured. There were eight patients with hypertension, three patients with diabetes, and three patients with both diabetes and hypertension as causes for CKD. Three of the CKDu patients had asthma as a chronic illness. Majority of the patient population (87.5%) were involved in farming either directly or assisting. None of the healthy individuals from both the endemic as well as non-endemic areas who volunteered for the study were involved directly in or assisted with farming.

**Table 3:** Characteristics of study population

	Total (*n* = 60)	Early Stage CKDu (*n* = 11)	Late Stage CKDu (*n* = 23)	Early Stage CKD (*n* = 5)	Late Stage CKD (*n* = 9)	Girandurukotte Healthy (*n* = 5)	Kandy Healthy (*n* = 7)
Age	51	52	54	53	60	35	34
	±12	±5	±8	±17	±6	±8	±9
Gender							
Male	51	9	19	4	9	4	6
Female	9	2	4	1	0	1	1
Other Chronic Diseases							
Hypertension	8	0	0	3	5	0	0
Diabetes	3	0	0	1	2	0	0
Hypertension + Diabetes	3	0	0	1	2	0	0
Asthma	3	2	1	0	0	0	0
Serum Cr	3.28 ± 2.20	1.62 ± 0.74	4.03 ± 2.51	1.28 ± 0.45	3.72 ± 1.36	-	-
eGFR	30.81 ± 21.15	52.32 ± 22.64	21.73 ± 11.09	64.61 ± 21.36	20.27 ± 7.50	-	-

### Gene expression analysis

**Fig. 1:**
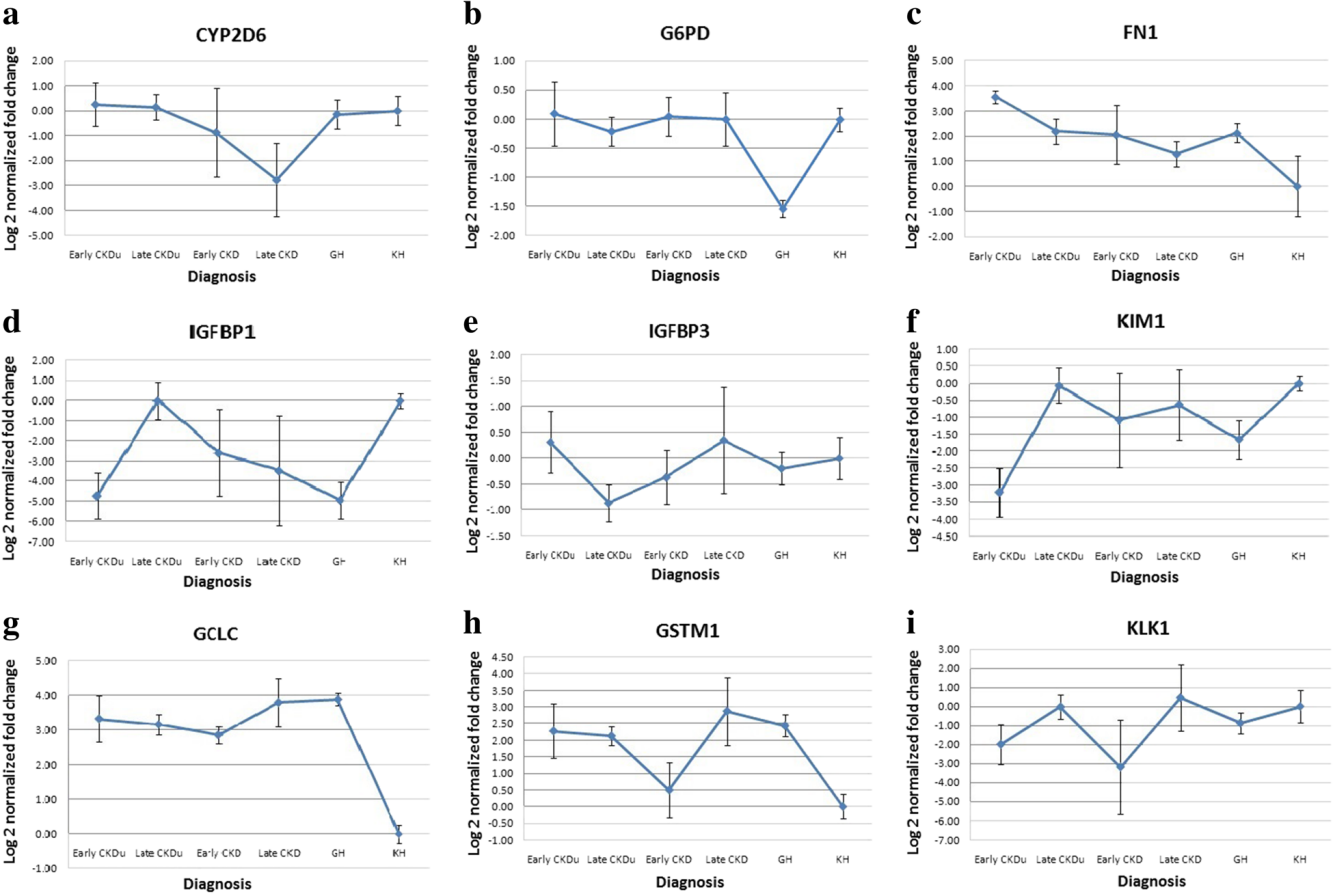
Group means with standard errors of log normalized gene expressions of **a** CYP2D6; **b** G6PD; **c** FN1; **d** IGFBP1; **e** IGFBP3; **f** KIM1; **g** GCLC; **h** GSTM1; **i** KLK1

The expressions of the selected genes were analyzed in the patient groups and the GH group using the KH group as calibrators. Group means with standard errors of log normalized values have been graphically presented (Fig. 1).

Significant differences were seen on one-way ANOVA, for four of the studied genes, IGFBP1 (F_5,52_ = 3.705, *p* = 0.007), KIM1 (F_5,56_ = 3.701, *p* = 0.006), GCLC (F_5,52_ = 5.535, *p* = 0.000), GSTM1 (F_5, 36_ = 3.143, *p* = 0.021) and KLK1 (F_5, 55_ = 2.925, *p* = 0.022). Post hoc Games-Howell test, equal variances not assumed, revealed significant difference between the KH group and early stage CKDu for both the IGFBP1 gene (*p* = 0.012) and the KIM1 gene (*p* = 0.003), and KH group and late stage CKDu and late stage CKD for the GSTM1 gene (*p* = 0.021 and *p* = 0.030). Significant difference was seen between the early and late stage CKDu for the IGFBP1 and the KIM1 genes (*p* = 0.010 and *p* = 0.006 respectively). The GCLC gene showed significant differences in expression between the KH group and all the other study groups (*p* < 0.05). The GH population was significantly different from the KH population for the three oxidative stress related genes G6PD (*p* = 0.004), GCLC (*p* = 0.004) and GSTM1 (*p* = 0.015), and also the KIM1 gene (*p* = 0.003). Significant difference between GH and late stage CKDu was seen in the G6PD (*p* = 0.007) and IGFBP1 (*p* = 0.013) genes.

Other significant results include absence of the GSTM1 gene expression in 11/34 CKDu patients (32.35%), 6/14 CKD patients (42.86%), 0/5 GH population and 3/7 KH individuals (42.86%).

### Correlation with currently used biomarkers

**Fig. 2:**
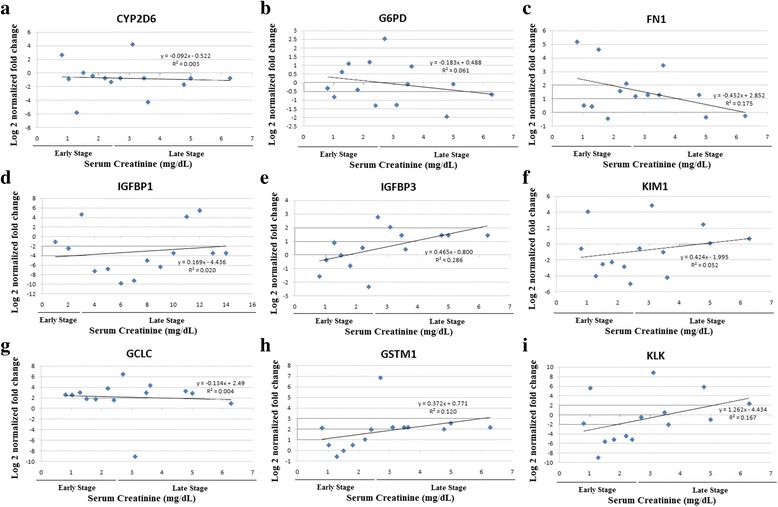
Correlation graphs of serum creatinine with log normalized gene expressions of **a** CYP2D6; **b** G6PD, **c** FN1; **d** IGFBP1; **e** IGFBP3; **f** KIM1; **g** GCLC; **h** GSTM1; **i** KLK1 in CKD patients

**Fig. 3:**
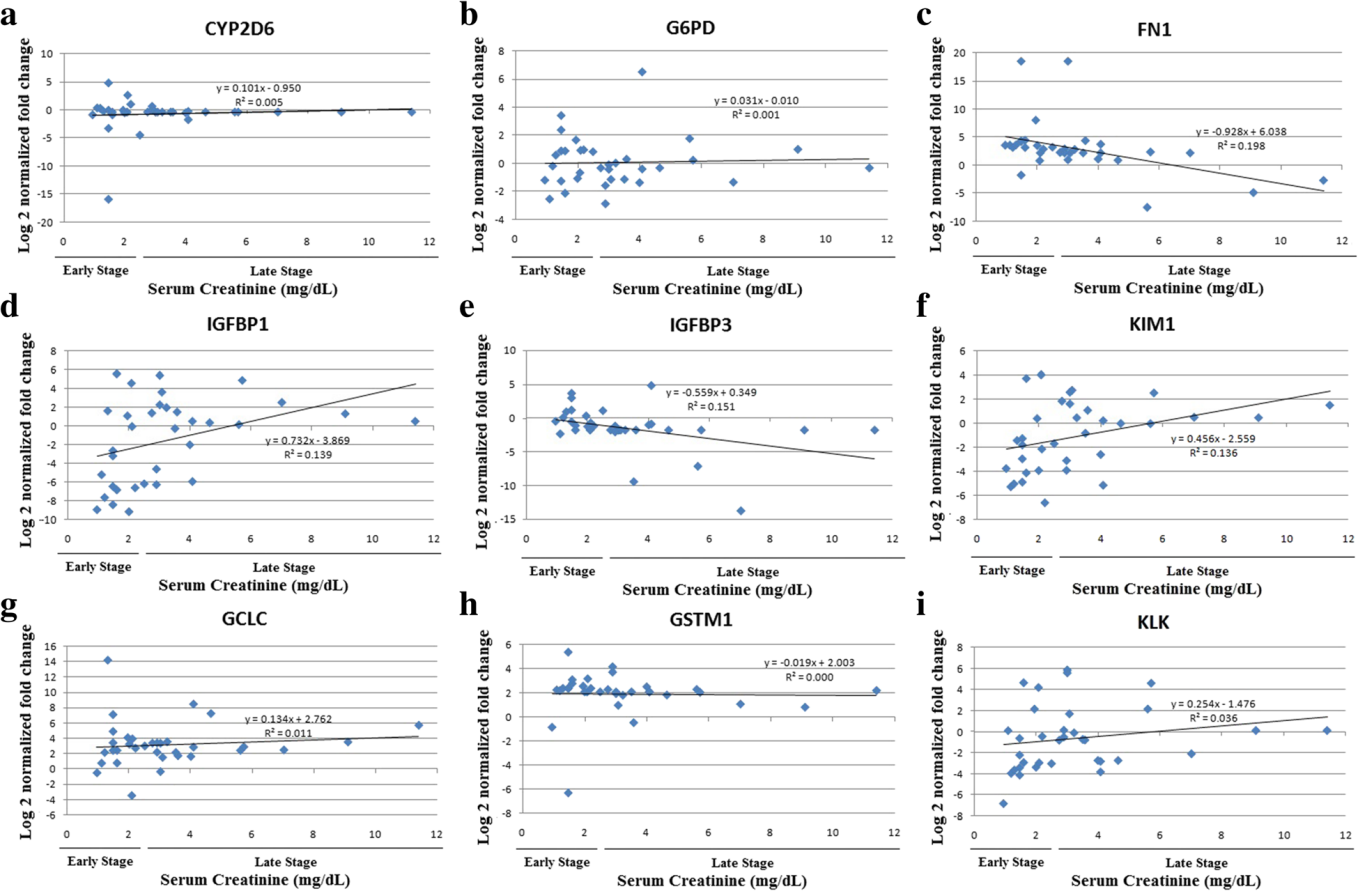
Correlation graphs of serum creatinine with log normalized gene expressions of **a** CYP2D6; **b** G6PD, **c** FN1; **d** IGFBP1; **e** IGFBP3; **f** KIM1; **g** GCLC; **h** GSTM1; **i** KLK1 in CKDu patients

The log normalized gene expression was correlated with two currently used markers of CKD; serum creatinine and eGFR, separately in the CKD and CKDu groups. Pearson correlation coefficients and two-tailed significance values were calculated (Table 4; Table 5), and correlation graphs plotted (Figs. 2, 3) respectively for CKD and CKDu. No significant correlations were seen in the CKD group in between the study genes and serum creatinine. In the CKDu group, FN1 and IGFBP3 had negative correlations with serum creatinine (*r* = −0.445, *p* = 0.008 and *r* = −0.389, *p* = 0.023 respectively) whereas KIM1 and IGFBP1 showed positive correlations (*r* = 0.369, *p* = 0.032 and *r* = 0.373, *p* = 0.030 respectively) with serum creatinine. IGFBP1, KIM1 and KLK1 showed negative correlations with eGFR (*r* = −0.513, *p* = 0.002; *r* = −0.443, *p* = 0.009; *r* = −0.340, *p* = 0.049 respectively). The other genes did not show any correlation of significance. In the CKD group, none of the genes showed any significant correlation with serum creatinine or eGFR.

**Table 4:** Correlation of log normalized gene fold changes with established biomarkers, serum creatinine and estimated GFR in the CKD group

		CYP2D6	G6PD	FN1	IGFBP1	IGFBP3	KIM1	GCLC	GSTM1	KLK	SrCr	eGFR
SrCr	Pearson Correlation	0.156	−0.491	−0.354	0.233	0.438	0.369	−0.383	0.280	0.515	1.000	−0.858^a^
	Sig. (2-tailed)	0.594	0.075	0.215	0.424	0.118	0.194	0.176	0.333	0.059	-	0.000
	N	14	14	14	14	14	14	14	14	14	14	14
eGFR	Pearson Correlation	−0.028	0.179	0.405	0.109	−0.472	−0.062	0.235	−0.386	−0.280	−0.858^a^	1.000
	Sig. (2-tailed)	0.925	0.540	0.151	0.711	0.088	0.833	0.419	0.173	0.333	0.000	-
	N	14	14	14	14	14	14	14	14	14	14	14

^a^Correlation is significant at the 0.01 level (2-tailed)

**Table 5:** Correlation of log normalized gene fold changes with established biomarkers, serum creatinine and estimated GFR in the CKDu group

		CYP2D6	G6PD	FN1	IGFBP1	IGFBP3	KIM1	GCLC	GSTM1	KLK	SrCr	eGFR
SrCr	Pearson Correlation	0.077	0.041	**−0.445** ^a^	**0.373** ^b^	**−0.389** ^b^	**.0369** ^b^	0.105	−0.024	0.190	1.000	**−0.743** ^a^
	Sig. (2-tailed)	0.666	0.820	**0.008**	**0.030**	**0.023**	**0.032**	0.554	0.891	0.282	-	**0.000**
	N	34	34	**34**	**34**	**34**	**34**	34	34	34	34	**34**
eGFR	Pearson Correlation	−0.141	−0.055	0.320	**−0.513** ^a^	0.408^*^	**−0.443** ^a^	−0.110	−0.150	**−0.340** ^b^	**−0.743** ^a^	1.000
	Sig. (2-tailed)	0.426	0.758	0.065	**0.002**	0.017	**0.009**	0.536	0.396	**0.049**	**0.000**	-
	N	34	34	34	**34**	34	**34**	34	34	**34**	**34**	34

^a^Correlation is significant at the 0.01 level (2-tailed)

^b^Correlation is significant at the 0.05 level (2-tailed)

All entries in boldface are significant at least at the 0.05 level

There was no statistical difference seen in the two-tailed ttest between the male and female populations within each study group.

## Discussion

We analyzed the expression patterns of a selected panel of genes in CKDu patients to test their possible use as early screening and prognostic biomarkers. In an era where personalized medicine is taking precedence over generalized diagnosis and treatment, the use of personalized biomarkers to identify specific molecular changes occurring in an individual is becoming more important [37, 38]. Although personalization to the individual level would not be financially feasible for developing countries, it is necessary to at least get specific to the disease level. For a disease like CKD, although there are some common pathways that lead to the progression of the disease, there are various primary causes having different pathophysiological mechanisms [2] and it would be advantageous to have biomarkers personalized to identify, monitor and treat them specifically.

This need is further realized in CKDu, a disease which is limited only to specific populations in certain geographic locations of the world. Although biomarkers of general CKD such as proteinuria, serum creatinine and GFR are currently being used in the diagnosis and prognosis of CKDu [10], this disease is believed to have a different, unidentified etiology, and therefore should give rise to different molecular level changes in the individual leading to the disease. In a geographical location endemic to CKDu, where individuals are exposed to similar environments as well as may be having diagnoses of other metabolic diseases, it can be easy to misdiagnose patients using common biomarkers and this brings a necessity to identify biomarkers to differentiate the groups from each other while also separating them from healthy individuals.

The symptoms of CKD usually do not become apparent till significant reduction of the kidney function has occurred. Stages 1 to 3 of the disease have been classified as early stage because of their asymptomatic progression and due to the fact that progression of the disease can be altered and complications reduced if identified during these stages [39]. Stages 4 and 5 have extensive kidney damage and usually lead to end-stage renal failure and therefore were considered as late stages of the disease.

In this study, analysis of gene expression was made using healthy population from Kandy, Sri Lanka; an area not endemic to the disease, as calibrators. These individuals belong to the same ethnicity and race, however were not residing in the same environment and were not involved in farming as an occupation. The study population had more males than females. The increased risk and incidence of CKDu in males has also been documented by other epidemiological studies [10, 11]. Any potential biomarker identified was expected to be significantly differentially expressed in the diseased groups. A healthy group from the CKDu endemic area, Girandurukotte, was also included in the study. As the etiology for the disease is believed to be environmental related, this population is also possibly exposed to the same disease causing factor/s and have to be considered a population at risk of developing disease. The gene expression patterns of this group were also thus compared to the healthy group from Kandy.

The fold changes observed in the CKD groups, both early and late stages, had high variation and therefore high standard errors as seen in Fig. 1. This could be due to the limited size of the CKD study population (*n* = 14) and the different etiologies for CKD within our study group, including hypertension (*n* = 8), diabetes (*n* = 3), and both (*n* = 3). The gene expression patterns of the CKD group also did not show any correlation with the currently used markers of the disease, serum creatinine and eGFR.

The CKDu group however showed lower standard errors and had fold changes of significant difference to the Kandy healthy group for some of the genes. Significant difference was observed between the KH group and early stage CKDu for the gene IGFBP1 (*p* = 0.012). IGFBP1 levels in human plasma has shown dynamic metabolic regulation [40]. Of the IGF binding proteins, IGFBP1 has been found to be the most regulated due to its acute down regulation by insulin and up regulation by glucoregulatory hormones and cytokines [16]. This gene is the main regulator of IGF-1 bioactivity and it has been found to play a major role in the development of diabetes and associated complications [41]. Low circulating serum levels of this enzyme has been associated with type 2 diabetes (T2D) in studies conducted in Swedish population [42–44] as well as in an Indian population [45]. Although our CKDu population was not diagnosed with diabetes, the IGFBP1 was most down regulated in the early stage CKDu, more so even than the CKD groups.

A similar difference was also observed between the KH group and early stage CKDu for the KIM1 gene (*p* = 0.003). The KIM1 gene was seen down regulated in the early stage CKDu patients when compared to the KH group. The KIM1 protein has been extensively studied and has been identified as a new specific marker for proximal tubule injury [46]. The protein and the gene have however been studied mainly in urine and kidney tissue biopsies, where it has always been up regulated with kidney disease [46–48].

Our finding in blood tissue is contrasting where it was seen down regulated in most of the diseased patients, with a highly significant decrease seen in the early stage CKDu patients. The role of KIM1 gene in blood is different to that of the kidney. The gene shares different names including Hepatitis A virus cellular receptor 1 (HAVCRI) and T-cell immunoglobulin and mucin domain (TIM1) [49, 50], owing to their different functions. Their functions mainly include coding for membrane receptors and associated with inflammatory healing response and infection [50]. Although it is difficult to assume the importance or the physiological response of this gene in blood of these diseased patients, the significant decrease seen in the early stage of CKDu indicates its potential as an early marker of the disease which needs to be further tested.

Significant differences in between the healthy populations from the two areas were seen for the G6PD, GCLC, GSTM1 and KIM1 genes, where the GH population showed similar expression patterns to that of the disease groups for the GCLC, GSTM1 and KIM1 genes. The healthy individuals of the GH group were those not diagnosed with CKDu on routine screening of population. However, as the initial stages of disease do not show any symptoms and there is a lack of sensitive early markers, the individuals from the GH group could be in the very early stages of disease formation, being residents of the high risk area. Follow up of these individuals and clinical testing could help answer these questions. If these individuals do indeed progress to disease formation, these genes would hold high potential as early diagnostic biomarkers.

Up regulation of the GCLC and GSTM1 genes in the Girandurukotte healthy population compared to Kandy healthy population has been documented in a separate cohort [51] and has been hypothesized to be due to the environmental oxidative stressors that the population may be exposed to. These genes therefore hold potential as early screening markers, but needs further follow-up and testing.

When correlating gene expressions with the existing markers of CKD/CKDu, FN1 and IGFBP3 had negative correlations with serum creatinine whereas KIM1 and IGFBP1 showed positive correlations. IGFBP1, KIM1 and KLK1 showed negative correlations with eGFR. FN1 gene showed a significant negative correlation with serum creatinine in the CKDu patients. This gene was up regulated in all the disease patients but was seen to be more highly expressed in the early stage CKDu than late stage CKDu, and early stage CKD than late stage CKD. The correlation of the gene expressions with the current biomarkers of kidney diseases further strengthens their potential use as biomarkers of CKDu.

The significant correlations of FN1, IGFBP1, IGFBP3 and KIM1 with serum creatinine and IGFBP1, KIM1 and KLK1 with eGFR in the CKDu patients indicates that these genes have a contributory factor to disease progression in this study group. Decrease of KIM1 and IGFBP1 expressions was seen in early stages of the disease with levels rising back to near Kandy healthy with later stages. Girandurukotte healthy individuals were also seen to have decreased expression of these genes similar to the early stages of disease (Fig. 1). It could be therefore postulated that the reduced baseline expression of these genes relates to disease susceptibility in this endemic population. The KIM1 and IGFBP1 gene also showed statistically significant difference in expression between the early and late stages of CKDu in the one-way ANOVA test further strengthening their possibility as prognostic biomarkers of CKDu.

The serum creatinine values were not obtained from the healthy individuals and therefore the correlation was based on values obtained only from disease patients. The expressions of the genes did not seem to have a steady progression with increasing severity of the disease as is seen with the established biomarkers. Drastic differential regulation was seen in the early stage CKDu compared to the KH group and the fold changes were near normal in the later stages of CKDu as seen in FN1, IGFBP1 and KIM1 genes. The expression of these genes from healthy through the increasing stages may not therefore be linear as in the case of serum creatinine, and the correlations seen may not indicate a relationship between the parameters, but individually regulatory mechanisms. For example, FN1 expression in blood has been associated with hypertension where increased expression was seen in hypertensive patients, being involved in protective mechanisms that limit organ damage [52]. The increased expression in early stage CKDu could be an initial protective effect which again diminishes with disease progression.

As a single biomarker will not suffice for clear identification of this disease with multiple possible etiologies and comorbidities, a panel of markers will have to be used. IGFBP1 and KIM1 genes have potential as early screening markers of CKDu as they showed significant differences in expression with late stage CKDu and the KH group. Increased expression of GCLC could be an indicator of environmental oxidative stress and the lack of GSTM1 gene is an indicator of increased susceptibility to oxidative stress. IGFBP1, IGFBP3, FN1 and KIM1 showed correlations with serum creatinine whereas IGFBP1, KIM1 and KLK1 showed correlations with eGFR therefore holding potential as progressive biomarkers of CKDu.

### Limitations of study

The study was a preliminary one looking at the possibility of selected genes as genetic biomarkers of CKDu. The study was carried out with a limited sample size for the identification of possible biomarkers with significant variances, which could then be validated in further studies with larger study populations. The healthy and disease groups belonged to different age categories which could be an influencing factor to the results.

## Conclusion

A panel consisting of IGFBP1, KIM1, GCLC and GSTM1 genes could be used in combination for early screening of CKDu, whereas these genes in addition with FN1, IGFBP3 and KLK1 could be used to monitor progression of CKDu. This is however a pilot study and the regulation of these genes have to be studied on larger scale populations to identify robust biomarkers that could be further tested for clinical use.

## Abbreviations

AKI: Acute kidney injury
ANOVA: Analysis of variance
cDNA: Complementary DNA
CKD: Chronic kidney disease
CKDu: Chronic kidney disease of unknown etiology
Ct: Threshold cycle
CYP2C19: Cytochrome P450 2C19
CYP2D6: Cytochrome P450 2D6
eGFR: Estimated glomerular filtration rate
FN1: Fibronectin-1
G6PD: Glucose-6-phosphate dehydrogenase
GCLC: Glutamate cysteine ligase C subunit
GFR: Glomerular filtration rate
GH: Girandurukotte Healthy
GSH: Glutathione
GSTM1: Glutathione-S-transferase mu 1
HAVCR1: Hepatitis A virus cellular receptor 1
IGF I: Insulin like growth factor 1
IGF II: Insulin like growth factor II
IGFBP1: Insulin like growth factor binding protein-1
IGFBP3: Insulin like growth factor binding protein-3
IQR: Inter-quartile range
KH: Kandy Healthy
KIM1: Kidney injury molecule-1
KLK1: Kallikrein-1
NADPH: Nicotinamide adenine dinucleotide phosphate
PCR: Polymerase chain reaction
SD: Standard deviation
T2D: Type 2 diabetes
TIM1: T-cell immunoglobulin and mucin domain
